# What nurses must understand about the ethics of assisted dying

**DOI:** 10.1002/nop2.2129

**Published:** 2024-03-16

**Authors:** Esme D. West, Tiffany N. Ricks

**Affiliations:** ^1^ CommUnityCare Health Centers Austin Texas USA; ^2^ Austin Community College Austin Texas USA

Assisted dying is gaining recognition as a treatment option for patients with a variety of illnesses. It is currently legal—in different forms and with varying levels of accessibility—in Austria, Belgium, Canada, Colombia, Germany, Italy, Luxembourg, the Netherlands, New Zealand, Portugal, Spain, Switzerland and parts of Australia and the United States (British Medical Association, 2023; Mroz et al., 2021; Roberts, 2023). Not only an option for those with terminal medical illnesses, assisted dying can also apply to chronic conditions and even to psychiatric disorders. While developments in healthcare and technology have advanced the ability to prolong and improve patients' lives, improving the dying process remains an understudied topic. Part of the problem is the ethical complexity surrounding assisted dying, which often leads to a lack of acceptance among physicians, healthcare systems and lawmakers. However, assisted dying is an important treatment that deserves to be included in a complete continuum of care. Hence, it is imperative that nurses and other healthcare professionals inform themselves about the ethical principles that support its relevance and validity.

It is helpful to start by defining key, related terms. *Physician‐assisted dying* refers to a physician aiding a person to die by providing medications for self‐administration so the patient may end their own life. This is in contrast to *euthanasia*, which refers to a physician (or nurse) administering medication to end the patient's life. Euthanasia may be *voluntary* (with the patient's consent), *non‐voluntary* (without consent when the patient lacks capacity to consent) or *involuntary* (against the patient's will) (Fontalis et al., 2018). Assisted dying also sometimes overlaps with *palliative care*, the primary goal of which is to relieve suffering. Palliative sedation during withdrawal of life‐sustaining therapies is a legal and widely accepted end‐of‐life treatment that can hasten the dying process (Andersen et al., 2022).

A central source of discourse in the assisted dying conversation is the concept of futility. In critical care, the word futile refers to treatments that ‘cannot accomplish the intended physiologic goal’ and treatments that could be perceived as *potentially inappropriate* since they might have ‘some chance of accomplishing the effect sought by the patient, but clinicians believe that competing ethical considerations justify not providing them’ (Kon et al., 2016). Studies show that many healthcare workers, professional organizations, patients and families agree that life‐prolonging interventions are inappropriate when the patient will not survive outside the acute care setting or when the patient has irreversible severe neurologic injury (Kon et al., 2016). Futility can also apply to other specialties, including psychiatry. Some severely ill psychiatric patients experience long‐term refractory depression and suicidality or self‐harm behaviours that do not respond to treatment, and assisted dying may be an appropriate option to relieve suffering when other treatments are futile. While assisted dying for psychiatric reasons is traditionally considered taboo, research supports incorporating it into the spectrum of care for psychiatrically futile cases, which should include harm reduction, palliative care and assisted dying (Friesen, 2020; Kirby, 2021).

## 1 ETHICAL PRINCIPLES

The ethical principle most often discussed in relation to assisted dying is autonomy. For medical patients, autonomy means having the right to make informed decisions about their care, and one expression of that autonomy is a person's right to refuse life‐saving treatments (American Nurses Association, 2015; Fontalis et al., 2018). However, it remains debatable whether autonomy should extend to a person's right to life‐ending treatments. The law must balance autonomy against society's need for public safety and order, and it is for this reason that assisted dying remains an issue of contention among healthcare providers and legislators around the world. A second relevant principle to consider is justice, which indicates that every person should be treated equally and impartially. While some opponents of assisted dying worry that it could be used to disproportionately and coercively end the lives of certain marginalized groups, most people who pursue assisted dying under Oregon's *Death With Dignity Act* typically have higher‐than‐average incomes and education (Hedberg & New, 2017). Justice is an important principle to consider when discussing the use of assisted dying, as it should serve as the foundation of safeguards that must be in place to prevent coercion and to ensure that inequitable access to health care is not a deciding factor for patients (Fontalis et al., 2018). It further challenges clinicians to ensure that people receive the holistic healthcare that we are all entitled to.

Finally, beneficence and non‐maleficence—the central principles of the Hippocratic Oath—instruct healthcare providers to act in the best interest of the patient and prohibit doing harm to the patient (Fontalis et al., 2018). Medical schools place a heavy emphasis on these two principles, along with concern for professional liability and the reputation of the medical field, producing, perhaps inadvertently, a population of physicians who are ill‐prepared for and/or unenthusiastic about providing assisted dying care. However, due to the shift from a physician‐centered care model to a team‐based care model, there is more pressure from patients for physicians to offer assisted dying care, as it is gaining more recognition as a way to fulfill the duty of beneficence (Fontalis et al., 2018).

## 2 NURSING IMPLICATIONS

Understanding the ethical foundation of assisted dying could potentially enable nurses to better serve their patients and communities, as well as improve and advance the nursing profession. Nurses have the ability to help patients navigate the concepts of futility and potential inappropriateness of treatments because they are well‐equipped to teach about the distinctions between interventions having no chance, a miniscule chance or a good chance of working. Increased awareness and education could potentially help patients and families make informed end‐of‐life decisions and avoid unnecessary pain and suffering (Pesut et al., 2019). Nurses can also help to mitigate the ethical risks posed by assisted dying. If administered without proper safeguards, assisted dying could violate the principles of autonomy, justice, beneficence and non‐maleficence, but nurses who understand these risks can advocate for policies that will act as safeguards while promoting equity in assisted dying (Fontalis et al., 2018; Pesut et al., 2019).

There is a natural concern for the experiences of patients and families, but healthcare leaders must also understand the implications of how *nurses* themselves experience assisted dying. Studies show that nurses involved in assisted dying see it as an important and appropriate addition to their scope of practice and that nurses play a central role in the navigation and provision of end‐of‐life care. Furthermore, end‐of‐life care is emotionally taxing work that can have profound psychological and emotional impacts on nurses. Hence, there is a significant need for ongoing opportunities for nurses to speak openly and process their experiences with assisted dying (Beuthin et al., 2018; Pesut et al., 2019).

## 3 CONCLUSION

Equipped with an understanding of the ethical principles of autonomy, justice, beneficence and non‐maleficence, nurses and other healthcare professionals can begin to engage in informed, nuanced conversations about assisted dying. This treatment option has the potential to benefit patients by enabling them to fully exercise their autonomy, while also experiencing beneficence from their providers. However, it is crucial to implement robust safeguards to ensure the equitable provision of assisted dying. Providers and nurses must better understand and appreciate the intersections of hope, futility and potentially ineffective treatments in order to help patients and families understand their options during end‐of‐life care. Healthcare leaders must establish opportunities for clinicians to reflect and process their experiences with assisted dying. If these provisions are in place, assisted dying can become a standard, evidence‐based addition to the continuum of care around the world.

## CONFLICT OF INTEREST STATEMENT

The authors certify that they have no affiliations with or involvement in any organization or entity with any financial interest (e.g. stock ownership), or non‐financial interest discussed in this manuscript.
